# The Inhibition of Serum Amyloid A Protein Aggregation by a Five-Residue Peptidomimetic: Structural and Morphological Insights

**DOI:** 10.3390/molecules29215165

**Published:** 2024-10-31

**Authors:** Julia Witkowska, Sandra Skibiszewska, Paweł Wityk, Marcel Pilarski, Elżbieta Jankowska

**Affiliations:** 1 Faculty of Chemistry, University of Gdańsk, Wita Stwosza 63, 80-308 Gdańsk, Poland; 2 Faculty of Chemistry, Gdańsk University of Technology, Narutowicza 11/12, 80-222 Gdańsk, Poland; 3 Fahrenheit Biobank, Medical University of Gdańsk, Dębinki 7, 80-211 Gdańsk, Poland

**Keywords:** serum amyloid A, aggregation, inhibitor

## Abstract

Serum amyloid A (SAA) is a small protein consisting of 104 residues and, under physiological conditions, exists mainly in hexameric form. It belongs to the positive acute-phase proteins, which means that its plasma concentration increases rapidly in response to injury, inflammation, and infection. The accumulation of SAA molecules promotes the formation of amyloid aggregates, which deposit extracellularly in many organs, causing their dysfunction. In our previous work, we successfully designed a peptidomimetic that inhibited the aggregation of amyloidogenic SAA fragments. In the present paper, we show how the same inhibitor, named saa3Dip, affects the oligomerization and aggregation processes of MetSAA1.1 protein. The thioflavin T assay showed that saa3Dip inhibited its fibrillization. The measurement of the internal fluorophore fluorescence (Trp) showed differences that occurred in the tertiary structure of MetSAA1.1 in the presence of the inhibitor, which was also confirmed by CD spectra in the aromatic range. FTIR results suggested that saa3Dip could stabilize some fragments of the native structure of MetSAA1.1, which was confirmed by determining the melting temperature (Tm) of the protein–inhibitor complex. AFM images demonstrated that the presence of saa3Dip prevented the formation of large SAA aggregates. Our results suggest that saa3Dip stabilizes the native conformation of MetSAA1.1.

## 1. Introduction

Serum amyloid A protein (SAA1.1) is an acute-phase protein produced mainly by the liver under the influence of inflammatory cytokines (IL-6, IL-1, and TNF-α) in response to inflammation, infection, injury, or metabolic stress [1]. SAA1.1 has diverse functions, such as modulation of the immune response, leukocyte chemotaxis, tissue remodeling, and effects on lipid metabolism through the binding of high-density lipoproteins [2]. It can also act as a molecular sensor, signaling the presence of tissue damage or infection. Elevated levels of SAA1.1 are associated with many inflammatory diseases, such as rheumatoid arthritis, Crohn’s disease, cancer, and viral and bacterial infections [3]. During the acute inflammatory phase, protein concentrations can rise as much as a thousandfold relative to normal conditions [4,5,6]. A persistently high level of SAA in the plasma and specific tissues favors the formation of amyloid aggregates and is believed to be the prerequisite for the development of reactive amyloidosis [7]. Among the most common pathological consequences of SAA aggregation are renal amyloidosis, where AA amyloid deposition leads to proteinuria and, in the final stage, renal failure; liver and spleen damage due to amyloid deposition in them and the development of hepatomegaly/splenomegaly; and cardiac dysfunction when, due to amyloid deposition in the myocardium, the myocardium weakens and fails [8,9].

Under physiological conditions, SAA1.1 exists mainly as a hexamer formed by the interaction of two trimers, in which each monomer consists of a bundle of four α-helices [10,11,12,13]. In the SAA trimer, the subunits are packed head to tail and are stabilized mostly by numerous hydrogen bonds and salt bridges. However, substituting the residues that are responsible for contacts of monomers in the trimer with alanine did not disturb the oligomeric state of SAA [13]. Presumably, it is the hydrophobic interactions between the two trimers that play a major role in the formation of the hexametric structure. The alanine substitution of residues from the interface between the trimers Trp53, Ile65, Phe68, and Phe69 led to the strong aggregation of SAA1.1, demonstrating the important role of these residues in protein oligomerization.

The proposed mechanism of SAA1.1 aggregation involves the dissociation of the non-amyloidogenic native hexamer. The initially formed small oligomers assemble into intermediate aggregates, called prefibrillar structures, which then form mature amyloid fibrils [13,14,15]. Studies of various amyloidogenic peptides and proteins, such as Aβ peptide and α-synuclein, suggest that the conversion of α-helix to β-sheet in partially folded, soluble protein oligomers may be an early step in amyloid formation [16,17,18,19]. Studies of amyloid-forming proteins have shown that π-stacking interactions play a significant role in the molecular recognition and self-assembly processes that lead to amyloid formation [20,21,22]. One method of inhibiting the process of pathological aggregate formation is therefore to shield sites prone to aggregation. This approach has been successfully tested in the case of β-amyloid peptide and α-synuclein, whose aggregation was inhibited in vitro by non-aggregating short peptides derived from those regions of the amyloidogenic molecules that are directly involved in their self-association [23,24,25]. Additionally, our previous studies have shown that aromatic interactions are crucial for effective aggregation inhibition. The short, five-amino-acid peptides designed by us, which possess natural and unnatural aromatic residues in their sequence, proved to be effective in inhibiting aggregation of fragments 1–12 and 1–27 of the SAA1.1 protein, which are considered its most amyloidogenic regions [26,27]. In our current work, we described how one of the most effective inhibitors, the saa3Dip peptide (Arg-Ser-Dip-Phe-Ser-NH_2_, where Dip—β,β-diphenylalanine), affects the structure and stability of the recombinant SAA1.1 protein (MetSAA1.1), reducing its tendency to aggregate.

## 2. Results and Discussion

### 2.1. Characterization of Aggregation Process of MetSAA1.1

**Figure 1:**
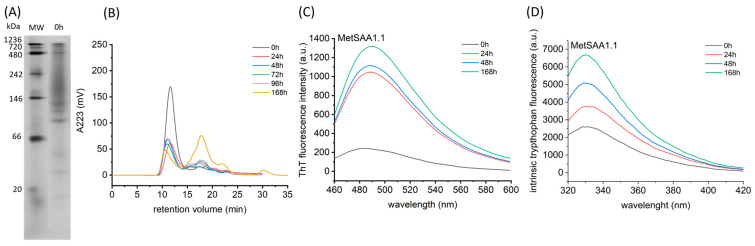
Characterization of aggregation process of MetSAA1.1. Native gel electrophoresis (**A**) and size exclusion chromatography (**B**) reveal the oligomerization profile of the protein at time point 0. C and D show aggregation progress during 7 days of incubation, characterized by thioflavin T assay (**C**) and the fluorescence of the internal fluorophore, tryptophan (**D**).

We started our research by studying the aggregation process of MetSAA1.1 in order to determine the appropriate incubation conditions for inhibitor testing. The electropherogram shown in Figure 1A clearly indicates that various oligomeric forms, both larger and smaller than the native hexamer, were already present in the protein sample at time point 0. This is likely to be the result of the initiated process of the dissociation of the native oligomer into dimers, trimers, and tetramers that may serve as the nuclei in the subsequent aggregation. This result was confirmed by size exclusion chromatography (SEC), which showed that with the extension of the sample incubation time, the number of smaller oligomers increased (Figure 1B). Initially, the increase was small, which seemed inconsistent with the dramatic decrease in the intensity of the main peak, corresponding to the native hexamer. We hypothesized that the smaller oligomeric forms were immediately consumed to form insoluble aggregates, which were discarded since only the supernatant obtained after the centrifugation of the incubated samples was used for chromatographic analysis.

To verify this hypothesis, we examined the aggregation process of MetSAA1.1 by measuring the fluorescence intensity of thioflavin T (ThT) added to the incubated samples at different time points (0, 24 h, 48 h, and 168 h). The graph shown in Figure 1C clearly demonstrates that already after 24 h incubation of the protein at 37 °C, the number of aggregates capable of interacting with this fluorescent probe increased significantly. Further changes were less intense, which agrees with the results of size exclusion chromatography.

The fluorescence of the internal fluorophore, tryptophan (Trp) provides information on what occurred in the tertiary and quaternary structures of MetSAA1.1 during its incubation. As can be seen in Figure 1D, as the incubation time increased, the fluorescence intensity of Trp also increased, which may indicate the structural transformation-induced distancing of Trp residue from intramolecular fluorescence quenchers. There are three Trp residues in the SAA protein, at positions 18, 53, and 85, so the signal is the resultant contribution of each. In the native hexameric conformation of the SAA protein, two of the residues, Trp18 and Trp85, were fully exposed to the solvent (Figure S1), and their fluorescence was largely quenched. Moving these residues into a more hydrophobic environment should be accompanied by changes in the position of the emission maximum, but this was not observed. Therefore, it is most likely that the increase in fluorescence intensity is contributed primarily by structural transformations involving the surroundings of the Trp53 residue. This residue in the native hexamer is located at the interface of the interacting trimers. Thus, the observed changes in fluorescence intensity may indicate that distancing of the trimers and/or rearrangement within their structure occurs during the aggregation process.

To investigate whether it is possible that changes in the Trp53 surroundings may be caused by the binding of the inhibitor, we analyzed the protein–inhibitor complex using AI on the AlphaFold Server [28]. We introduced the MetSAA1.1 sequence six times to potentially allow the creation of a hexamer. As a compound mimicking saa3Dip we introduced the RSFFS sequence, in which Dip is substituted with its natural analog, phenylalanine. The RSFFS is a parent compound of saa3Dip and has been confirmed to inhibit aggregation of the highly amyloidogenic fragment of human SAA1.1, SAA1-27 [26]. In the highest probability model, a hexamer with a structure almost identical to the experimental structure of the SAA protein (PDB code 4IP9) is indeed formed. The inhibitor binds in it between the two trimers and interacts with Trp53 from one trimer and Phe69 from the second one (Figure S2). These residues play an important role in the oligomerization of SAA [13]. We hypothesize that saa3Dip stabilizes the native hexamer by binding in a similar way; however, we are aware that the confidence score of the predicted structure is low.

### 2.2. Saa3Dip Effect on the Fibrillization of MetSAA1.1 Examined Using ThT Assay

**Figure 2:**
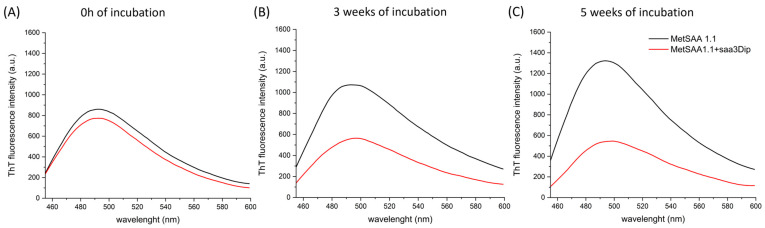
Representative plots showing the aggregation process of MetSAA1.1 alone and in the presence of saa3Dip inhibitor, monitored using the amyloid-binding dye ThT. Results are shown (**A**) at the starting point, (**B**) after 3 weeks, and (**C**) after 5 weeks of incubation at 37 °C.

We conducted aggregation studies on a commercially available recombinant protein, which has an additional methionine residue at the N-terminus. This choice was based on the report by Patke et al., which highlighted a significantly higher fibrillization potential of the MetSAA1.1 than that of the native isoform lacking the methionine residue [15]. The experiments performed showed, however, that the protein we purchased behaves differently from the protein that Patke et al. had at their disposal. First, the SEC analysis of their protein indicated that it mainly comprised a tetramer and some octamers and monomers, while the SEC chromatogram we present in Figure S3 clearly shows that our samples contained mainly a hexamer. Secondly, in contrast to their protein, which formed stable fibrillar aggregates already after 72 h of incubation, the aggregation process of our protein did not reach equilibrium during the 7-day incubation at 37 °C (Figure S4). Therefore, we investigated the influence of saa3Dip on the fibrillization of MetSAA1.1 during a five-week incubation. We chose such a long incubation time also because we wanted to assess whether the effect of the inhibitor did not fade over time. The progress of aggregation was monitored by using the ThT assay. As can be seen in Figure 2A and Figure S5A, the structures that were able to interact with ThT were present in both samples from the very beginning of the experiment (at time ‘0’). However, after 3 weeks of incubation, the number of aggregates in the samples of MetSAA1.1 alone increased, while in the samples with saa3Dip, the fibrilization process was inhibited (Figure 2B and Figure S5B). The inhibitory effect was retained after a five-week incubation (Figure 2C and Figure S5C). This confirms that saa3Dip is able to inhibit the fibrillization of MetSAA1.1 protein, as it did for its fragment, saa1-27 [27].

We checked that saa3Dip does not interact with ThT (Figure S6). Also, during the studies, we repeated ThT tests many times, using several batches of MetSAA1.1, purchased at different times. The aggregation of amyloidogenic proteins is a complicated process, and its mechanism is influenced by many factors; therefore, the rate of growth of MetSAA fibrillar structures was variable. Nevertheless, we constantly observed the same correlation—the gradual increase in ThT fluorescence intensity for the protein and a roughly constant fluorescence level for the protein samples incubated in the presence of the inhibitor, which indicated the inhibition of the aggregation in the presence of saa3Dip.

### 2.3. Morphology of MetSAA1.1 Oligomers/Aggregates in the Presence of saa3Dip

**Figure 3:**
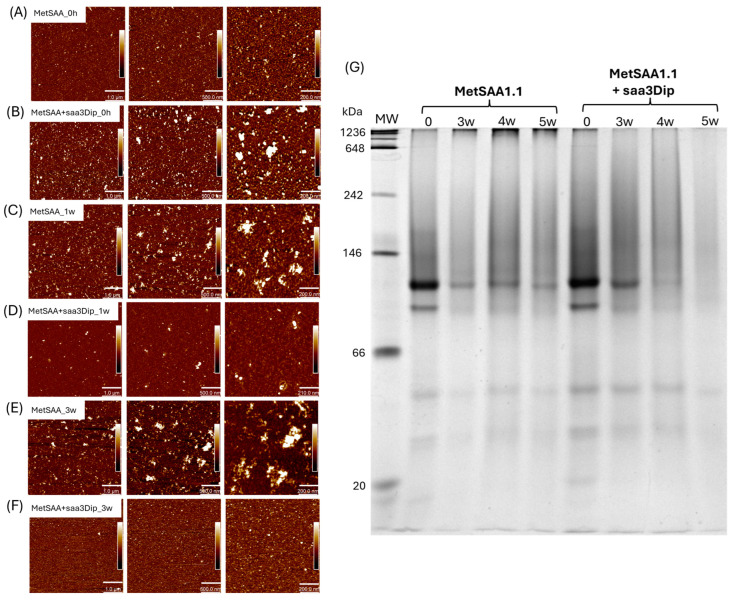
AFM images of MetSAA1.1 and MetSAA1.1+saa3Dip: (**A**,**B**) samples at the starting point of incubation; (**C**,**D**) samples after 1 week of incubation at 37 °C; (**E**,**F**) samples after 3 weeks of incubation at 37 °C. The individual panels from left to right show the same area but in increasing zoom; from left to right, the scale bar corresponds to 1 µm, 500 nm, and 200 nm, respectively; (**G**) electropherogram of native PAGE for MetSAA1.1 and MetSAA1.1+saa3Dip samples incubated for 0, 3, 4 and 5 weeks at 37 °C.

AFM images of MetSAA and MetSAA+saa3Dip demonstrate that, at the beginning of incubation (Figure 3A,B), both samples are quite homogeneous and contain oligomers of similar shape. However, in the presence of saa3Dip (Figure 3B), slightly larger structures can also be seen. They probably were due to the transient formation of clusters of the inhibitor molecules before they had time to interact with the protein. However, as can be inferred from the AFM images obtained for the samples after 1 and 3 weeks of incubation, these clusters disappeared over time, and the interaction of saa3Dip with the protein contributed to the inhibition of its aggregation. After 1 and 3 weeks of incubation at 37 °C (Figure 3C–F), a morphological difference between the oligomers is evident in both samples. Images of the MetSAA sample (Figure 3C,E) show aggregation progress; we can see large amorphous aggregates and smaller prefibrillary structures. At the same time, images of the protein incubated with saa3Dip do not show any large aggregates, and even the sample is more homogeneous than at time ‘0’ (Figure 3D,F). This result was confirmed by native electrophoresis (Figure 3G and Figure S7). In the paths corresponding to 3-, 4-, and 5-week incubation of the protein alone, large oligomeric structures can be seen in the upper part of the gel. These structures are unable to migrate in the gel. Their number increases with increasing incubation time. A smaller number of similar structures are visible for the ‘0’ samples of MetSAA and the protein incubated in the presence of saa3Dip. However, in the presence of the inhibitor, increasing the incubation time results in the disappearance of such structures, indicating the ability of saa3Dip to inhibit MetSAA aggregation.

As the gel indicates, oligomers of different sizes are already present in the MetSAA1.1 sample at the ‘0’ time point. As can be seen from the size exclusion chromatogram (Figure S3), the main form of the native protein is hexamer, which elutes at a volume close to the calibration protein of 66 kDa (molecular weight (MW) of the hexamer is 70.2 kDa). It is more difficult to assess the protein size based on the position of its band relative to the marker in native electrophoresis since in addition to the charge of the protein, its size and shape also influence the migrating properties. In the gel presented in Figure 3G, the most intense band is positioned between the 66 and 146 kDa markers, and although it migrates higher than one would expect, it most likely corresponds to the hexamer. Below that, another band is visible, which rather corresponds to a hexamer with a different conformation than a pentamer (MW 58.5 kDa). The next visible bands are most likely a tetramer (MW 46.8 kDa) and a trimer (35.1 kDa).

Surprisingly, there are almost no bands visible for MetSAA1.1+saa3Dip in the 5w line. However, on the gel, we run for the second set of samples (Figure S7), the bands in the 5w line are visible, although with lower intensity than for the time ‘0’ sample. The wells were not cut off from this gel before staining, so it can be observed that with increasing incubation time, aggregates were formed that did not have the ability to enter the gel. Most likely they are responsible for the lower intensity of the bands corresponding to the smaller oligomers. These aggregates appear both for the protein incubated alone and with the inhibitor, but in the latter case, the aggregates are noticeably fewer, confirming the ability of saa3Dip to inhibit MetSAA1.1 aggregation.

### 2.4. Inhibitor-Induced Changes in the MetSAA1.1 Structure Studied Using FTIR Spectroscopy

**Figure 4:**
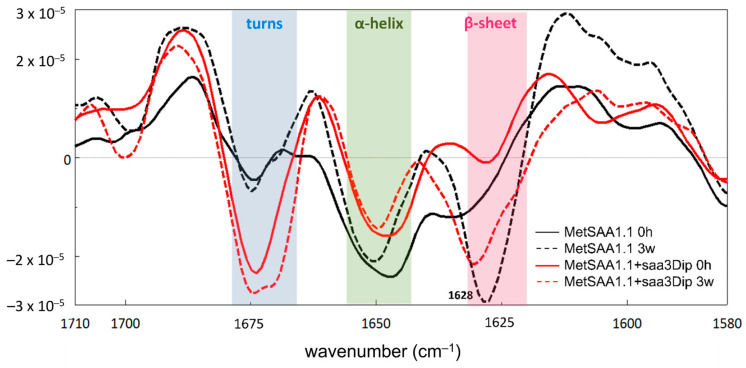
Second-derivative FTIR spectra of the amide I region of MetSAA1.1 (black line) and MetSAA1.1+saa3Dip (red line) at the starting point (solid line) and after 3 weeks of incubation at 37 °C (dashed line).

To observe whether any changes occur in the secondary structure of MetSAA upon saa3Dip binding, we used Fourier transform infrared spectroscopy (FTIR). The traces of the second derivative of the amide I band recorded for MetSAA alone (black) and for the protein in the presence of saa3Dip (red) are presented in Figure 4. The most significant differences caused by the incubation of the samples for 3 weeks at elevated temperatures occurred in the range 1620–1635 cm^−1^ (marked in pink). This region corresponds to the β-sheet conformation. For the β-strands found in amyloid structures, a shift in this band toward lower wavelengths is characteristic. Such a shift is visible in the case of MetSAA, which was incubated alone. The band at 1634 cm^−1^, present in the derivative trace at time ‘0’ (solid black line), shifts to 1628 cm^−1^ after 3 weeks of incubation (dashed black line). It is accompanied by a deepening of the minimum, indicating a greater contribution of the β-sheet to protein conformation. This conformational change is confirmed by a weaker minimum around 1650 cm^−1^, characteristic of an α-helix, which reflects a partial loss of the native MetSAA structure. Surprisingly, in light of the ThT assay results, the presence of the inhibitor does not dramatically affect the appearance of the β-sheet structure. In the case of MetSAA+saa3Dip samples, incubation at 37 °C also led to an increase in the amount of this conformation, as indicated by the deepened minimum (red dashed line) (Figure 4 and Figure S8). However, in the case of the protein–inhibitor sample, the band at 1670 cm^−1^, corresponding to turns, is much more pronounced. This minimum did not vary in position or depth during incubation, suggesting that the saa3Dip is able to stabilize some fragments of the native MetSAA structure.

### 2.5. Saa3Dip Effect on the Structure of MetSAA1.1 Studied by Means of CD and Trp Fluorescence

**Figure 5:**
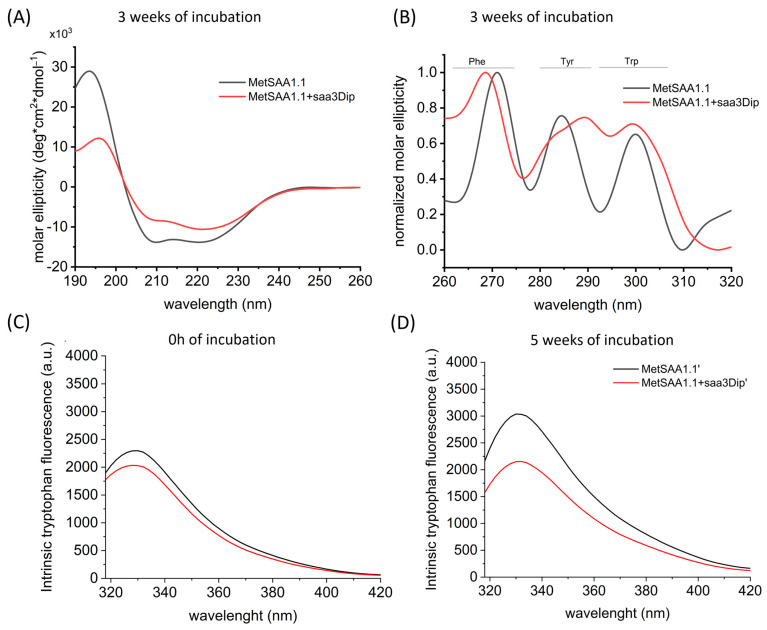
Far-UV (**A**) and near-UV (**B**) CD spectra of MetSAA1.1 and MetSAA1.1 with saa3Dip, incubated for 3 weeks at 37 °C. Representative spectra showing intrinsic Trp fluorescence of MetSAA1.1 and MetSAA1.1+saa3Dip (**C**) at the starting point and (**D**) after 5 weeks of incubation at 37 °C.

We also examined the effect of the inhibitor binding on protein conformation by recording circular dichroism (CD) spectra. The far-UV region (190–240 nm) corresponds to the peptide bond absorption and provides information about the secondary structure, while the near-UV range (260–320 nm) reflects the environment of the aromatic side chains and gives information about the tertiary structure of the protein. CD spectra were registered after the three-week incubation of MetSAA and MetSAA with saa3Dip. Since in this technique, only the supernatant can be used, the CD spectra reflect the conformation of the moieties present in the soluble fraction. As can be seen in Figure 5A, the far-UV spectra have the shape typical of an α-helix (positive maximum at 195 nm and two minima, at 209 and 223 nm) for both samples. Moreover, these spectra are identical to the spectrum of native MetSAA, recorded just after protein dissolution (Figure S9). This means that the secondary structure of the soluble oligomers does not change significantly compared to the native hexamer. For the near-UV traces, the situation is different (Figure 5B and Figure S10). The spectra of the protein and the protein treated with saa3Dip visibly differ, especially in the range showing the surroundings of tyrosine (Tyr) and Trp residues. The changes seen in this spectrum seem to confirm the involvement of aromatic residues in the aggregation process, which has already been reported for SAA and other proteins [29,30,31,32]. They also indicate that these very interactions are influenced by saa3Dip binding.

To further explore the involvement of aromatic residues in the MetSAA–saa3Dip interactions, we used intrinsic Trp fluorescence. At the starting point, the traces for the protein and the protein in the presence of saa3Dip followed a similar pattern and did not differ in intensity (Figure 5C and Figure S11A), as most likely the interaction between the molecules had not yet occurred. However, after 5 weeks of incubation, an increase in fluorescence intensity was evident in the sample with the protein alone, while for MetSAA+saa3Dipl it stayed almost at the same level (Figure 5D and Figure S11B). The results of this experiment confirmed that the surroundings of Trp residues are influenced by inhibitor binding. As we did not see the band shift toward shorter or longer wavelengths, the enhanced fluorescence was probably the consequence of changes in the position of Trp residues in relation to the intrinsic fluorescence quencher rather than changes in the polarity of the microenvironment surrounding the Trp fluorophore.

As we already mentioned, three Trp residues are present in MetSAA, only one of which, namely Trp53, is not exposed to solvent in the protein hexameric structure. In the native hexamer, this residue is located at the interface of the interacting trimers. Changes within this interface, in the vicinity of Trp53, most probably contribute to the increase in fluorescence observed for the aggregating protein. This suggests that the aggregation process leads to moving the trimers away from each other and/or rearranging them in a way that increases the distance between Trp and its quencher. On the other hand, saa3Dip seems to keep the trimers together as no changes in the fluorescence intensity were observed for samples containing the inhibitor.

### 2.6. Thermal Denaturation of MetSAA1.1 and Its Complex with saa3Dip

**Figure 6:**
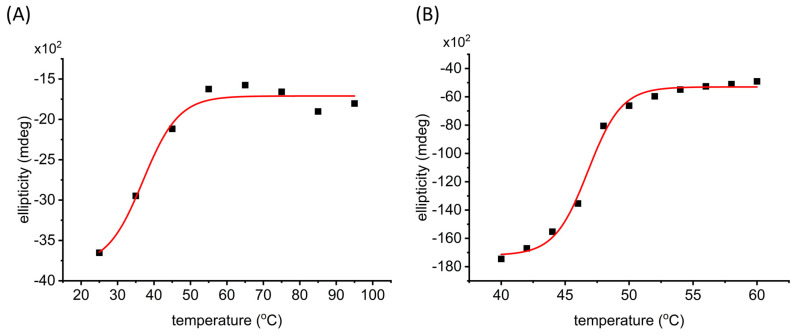
The thermal denaturation of MetSAA1.1 (**A**) and its complex with saa3Dip (**B**) observed using circular dichroism at λ = 222 nm.

Since our results indicate that saa3Dip can inhibit MetSAA aggregation by stabilizing its native conformation, we decided to perform thermal denaturation studies to check this hypothesis. Ellipticity at 222 nm was monitored as a function of temperature to assess if the inhibitor influences the stability of the native helical conformation. As can be observed in Figure 6, the melting temperature of the protein itself is about 40 °C. This result was confirmed by tests carried out using the Prometheus NT48 (NanoTemper Technologies GmbH, Munich, Germany), which utilizes advanced differential scanning fluorimetry to observe even minimal changes in Trp fluorescence (nanoDSF). These measurements indicated a value of 40.7 °C as the melting temperature of the protein itself (Figure S12). Unfortunately, we were not able to perform a similar analysis for MetSAA+saa3Dip. However, measurements using circular dichroism over a narrow temperature range conclusively proved that the melting temperature for the complex is several degrees higher. This confirms that saa3Dip stabilizes the native conformation of the protein, and probably through this, it inhibits its oligomerization/aggregation.

The thermal stability of our MetSAA1.1 was about 10 °C higher than previously described [15]. The increased stability is correlated with a reduction in fibrillization potential. We hypothesize that it could be caused by different oligomeric states of our protein and the protein examined by Patke et al. [15]. In our experiments, MetSAA1.1 mainly formed a hexamer, which is considered a native oligomer, while in their research, Patke et al. used a protein comprising tetramers, octamers, and monomers, which may indicate that structural transformations of the native oligomer had already started, and for this reason, the protein showed high fibrillation potential.

## 3. Conclusions

Our studies demonstrate that saa3Dip is an effective inhibitor of MetSAA1.1 protein aggregation preventing the formation of its large aggregates. The proposed mechanism of SAA aggregation involves the dissociation of native oligomers. We confirmed this by showing that SAA aggregates are not formed by native monomers/oligomers, but their formation is preceded by structural transformations. The results of structural studies suggest that saa3Dip stabilizes some elements of the native structure of the MetSAA1.1 protein, and thermal denaturation analysis shows that, in the presence of the inhibitor, the melting point of the protein is several degrees higher. This suggests that saa3Dip stabilizes native forms of the protein and probably through this inhibits oligomerization/aggregation processes.

## 4. Materials and Methods

General Procedures: All reagents and solvents were obtained from commercial sources and used without further purification. MetSAA1.1 was purchased from PeproTech (Cranbury, NJ, USA; #300-53). Different lots of the protein were used in the studies. For all experiments, the incubation conditions were the same. Samples were prepared in Tris-buffered saline (20 mM Tris + 200 mM NaCl; TBS) at pH 7.4, in tightly closed LoBind Eppendorf tubes. Unless otherwise noted, the protein concentration was 1 mg/mL (0.85 μM), and saa3Dip was added in a 12-fold molar excess over protein. The samples were incubated for up to 5 weeks in a thermoshaker set at 300 rpm and 37 °C.

Synthesis of saa3Dip: The peptide was synthesized on a solid support (TentaGel R RAM) according to standard 9-fluorenylmethoxycarbonyl (Fmoc) chemistry, using a Liberty Blue microwave peptide synthesizer (CEM, Matthews, NC, USA). The coupling of the Fmoc-protected amino acids was carried out utilizing a 1:1 mixture of 0.5 M N,N′-diisopropylcarbodiimide (DIC) and 1 M ethyl cyano(hydroxyimino)acetate (Oxyma Pure) in dimethylformamide. The purification of the crude product was performed by semipreparative reverse-phase high-performance liquid chromatography using a Jupiter 4 µm Proteo column, 90 Å, 21.2 mm × 250 mm, 15 mL/min, 60 min gradient from 10% to 80% aqueous acetonitrile containing 0.1% TFA. The identity of the pure product was evaluated by MALDI-TOF MS (Biflex III, Bruker, Billerica, MA, USA).

Intrinsic Trp fluorescence spectroscopy: After incubation, 5 µL of MetSAA1.1 and MetSAA1.1+saa3Dip samples were transferred into 96-well U-shape black plates containing 95 µL of TBS buffer. Fluorescence was recorded at 300–420 nm. The excitation wavelength was set at 290 nm (Infinite M200 Pro; Tecan Trading AG, Männedorf, Switzerland).

ThT fluorescence measurement: ThT fluorescence was measured for the same samples as Trp fluorescence. Immediately before measurement, 6 µL of 500 µM aqueous ThT solution was added to each well. Fluorescence emission was recorded in the range 455–600 nm using Infinite M200 Pro. The excitation wavelength was set at 420 nm.

Native electrophoresis: Electrophoretic separations were carried out for about 5 h at 4 °C on a 10% native polyacrylamide gel. The applied voltage was 90 V. The electrode buffer consisted of 25 mM Tris and 192 mM glycine. Briefly, 9 μL of the sample supplemented with a native loading buffer was applied to each well. Gels were stained with Coomassie Blue.

Size-exclusion chromatography: The assays were performed on a Superdex 75 3.2/300 column using Omnisec chromatography unit (Malvern Panalytical, Malvern, United Kingdom). Samples dissolved in TBS buffer pH 7.4 were incubated for 24 h, 48 h, 72 h, 96 h, and 168 h at 37 °C with continuous shaking at 300 rpm. The same buffer was used for elution as for incubation of the samples. Before each injection, the samples were centrifuged, and 10 μL of the supernatant was applied to the column.

CD Spectroscopy: The CD spectra were recorded on a JASCO J-815 spectropolarimeter (JASCO Corporation, Tokyo, Japan) in the far-UV (190–260 nm) and near-UV region (260–320 nm) at room temperature. Immediately before measurement, samples were diluted with water to 0.2 mg/mL and centrifuged. Only the soluble fraction was tested.

ATR-FTIR spectroscopy: For FTIR analysis, samples of MetSAA1.1 and MetSAA1.1+saa3Dip were prepared similarly to other incubations, except that the protein concentration was increased to 2 mg/mL. The spectra were collected at a resolution of 2 cm^−1^ using IFS-66 spectrometer (Bruker Optics, Ettlingen, Germany). Then, 32 interferograms were averaged for each spectrum and baseline-corrected. The second derivative of absorbance was calculated for the amide I band region, using the Savitzky–Golay 5-point smoothing filter.

Atomic force microscopy (AFM): MetSAA1.1 and MetSAA1.1+saa3Dip were incubated at the protein concentration 2 mg/mL and then diluted to 1.6 μM by adding 20 mM Tris pH 7.4. AFM visualizations of aggregates were performed using BioScope Resolve AFM (Bruker, Bremen, Germany) at 23 °C. The ScanAsyst-Air probe (Bruker) was used for atomic force imaging. Images were taken at 512 × 512 pixels with a PeakForce Tapping frequency of 1 kHz and an amplitude of 150 nm. A height sensor signal was used to display the protein image using NanoScope Analysis v1.9.

Thermal denaturation: CD spectra were recorded on a JASCO J-815 spectropolarimeter. MetSAA1.1 and MetSAA1.1+saa3Dip samples with a protein concentration of 1 mg/mL were incubated at room temperature for one hour and then diluted with water to 0.2 mg/mL. The far-UV spectra (190–260 nm) were recorded in the temperature range either 25–95 °C in 10 °C steps (for MetSAA1.1) or 40–60 °C in 2 °C steps (for MetSAA1.1+saa3Dip). Before the spectrum acquisition, the sample was left for 1 min at each temperature.

## Data Availability

The research data will be made available upon request.

## Supplementary Materials

The following supporting information can be downloaded at: https://www.mdpi.com/article/10.3390/molecules29215165/s1, Supplementary Figures: S1–S12. Figure S1: Location of Trp residues in the hexameric structure of human SAA protein; Figure S2: Alpha Fold Server prediction of the site of interaction between MetSAA1.1 hexamer and RSFFS peptide; Figure S3: SEC chromatogram of MetSAA1.1 overlaid with chromatograms of calibrating proteins; Figure S4: ThT fluorescence intensity changes during the 7-day incubation of MetSAA1.1; Figure S5: Representative plots of ThT fluorescence of MetSAA1.1. and MetSAA1.1+saa3Dip samples incubated for 0h, 3 weeks and 5 weeks; Figure S6: Thioflavin T fluorescence in the presence of saa3Dip; Figure S7: Native PAGE electropherogram of MetSAA1.1 and MetSAA1.1+saa3Dip samples incubated for 0, 3, 4 and 5 weeks; Figure S8: Second-derivative of the FTIR spectrum of saa3Dip inhibitor; Figure S9: Far-UV CD spectrum of native MetSAA1.1; Figure S10: Near-UV CD spectrum of saa3Dip; Figure S11: Representative spectra showing intrinsic Trp fluorescence of MetSAA1.1 and MetSAA1.1+saa3Dip at the starting point and after 5 weeks of incubation; Figure S12: MetSAA1.1 stability observed using differential scanning fluorimetry.
