# Supplementary material for: The Inhibition of Serum Amyloid A Protein Aggregation by a Five-Residue Peptidomimetic: Structural and Morphological Insights
Source: Molecules. 2024 Oct 31;29(21):5165. doi: 10.3390/molecules29215165 (PMC11547336; doi:10.3390/molecules29215165)
Supplement: Supplementary file 1 [file molecules-29-05165-s001.zip › molecules-3257338-supplementary.pdf]

Supplementary materials

Inhibition of Serum Amyloid A Protein Aggregation by a Five-Residue Peptidomimetic: Structural and Morphological Insights

Julia Witkowska, Sandra Skibiszewska, Paweł Wityk, Marcel Pilarski, Elżbieta Jankowska

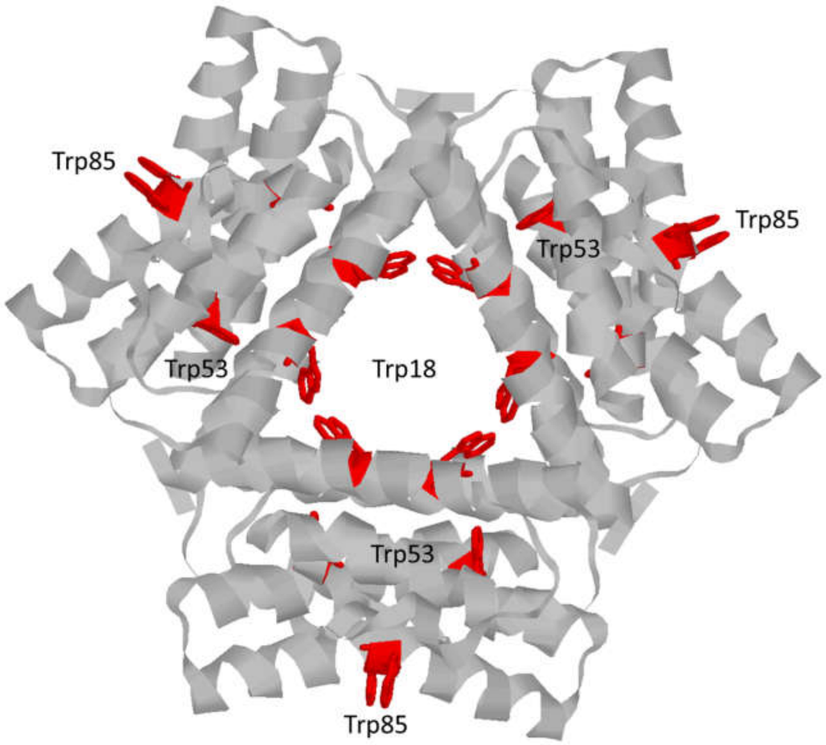

Figure S1. Structure of hexameric human serum amyloid A protein (PDB code 4IP8) with all tryptophan residues marked in red.

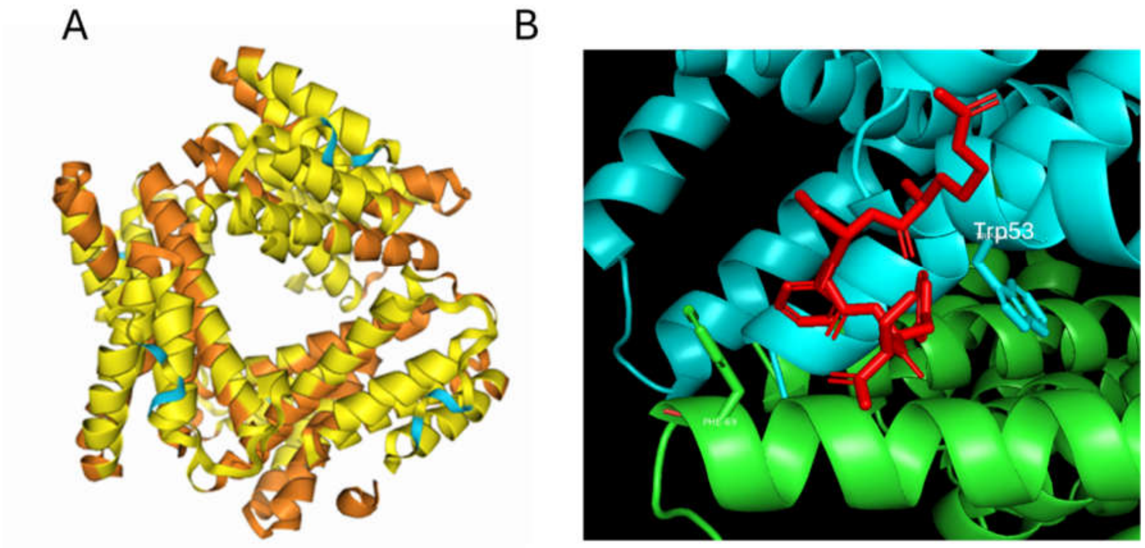

Figure S2. A. Alpha Fold Server prediction of interaction of MetSAA1.1 hexamer with RSFFS peptide (score: ipTM = 0,12; pTM = 0.21). B. Binding of RSFFS peptide with MetSAA1.1 hexamer in close proximity to Trp53.

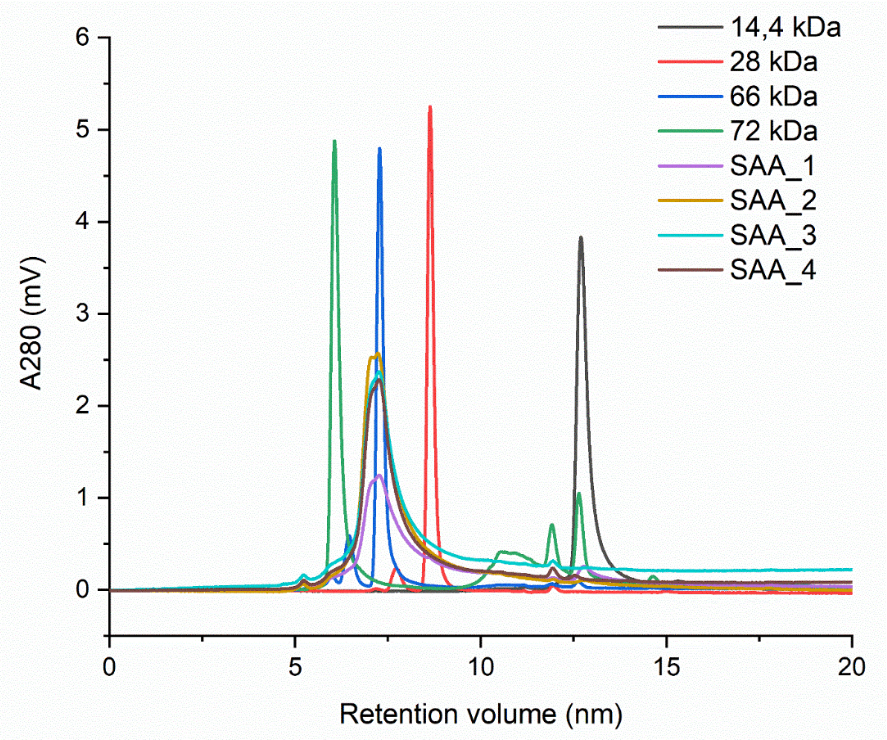

Figure S3. Size exclusion chromatograms of four different samples of MetSAA1.1 (SAA_1, SAA_2, SAA_3 and SAA_4) overlaid with the chromatograms of calibration proteins, lysozyme (MW 14.4 kDa), anhydrase (MW 28 kDa), bovine serum albumin (MW 66 kDa), and type IV collagenase (MW 72 kDa).

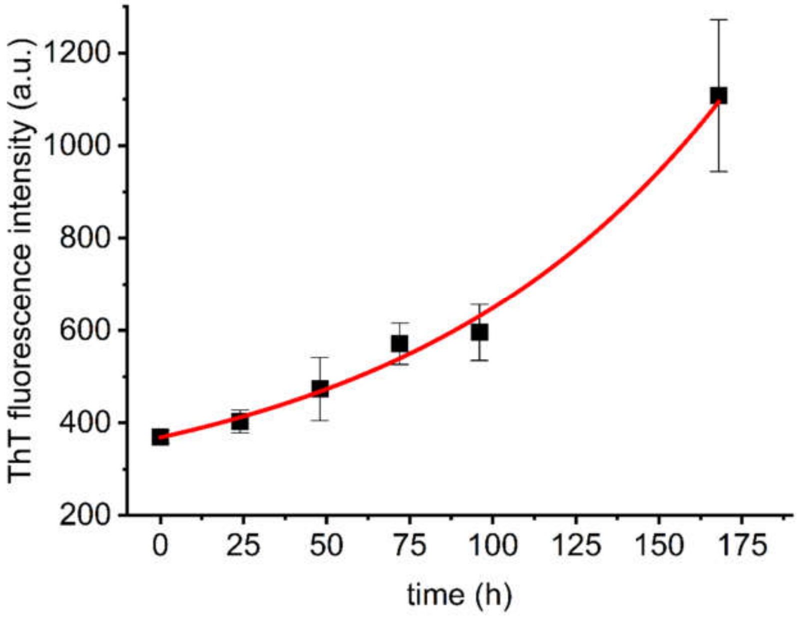

Figure S4. ThT fluorescence intensity observed during 7-day incubation of MetSAA1.1 at 37°C.

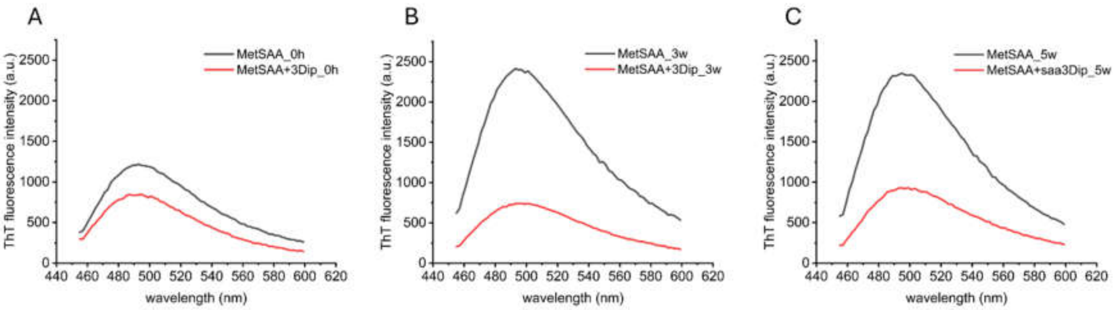

Figure S5. Representative plots showing aggregation process of MetSAA1.1 alone and in the presence of saa3Dip inhibitor, monitored using the amyloid-binding dye, ThT. Results are shown A) at the starting point, B) after 3 weeks, and C) after 5 weeks of incubation at 37°C.

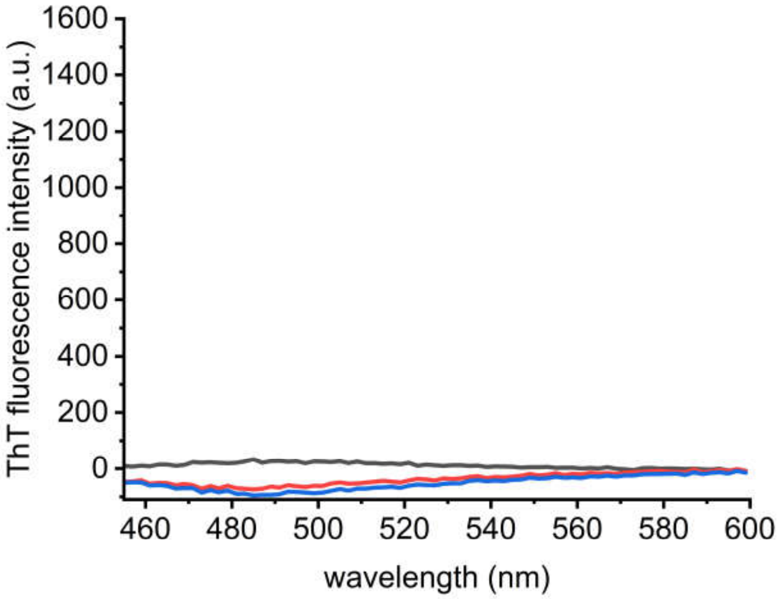

Figure S6. Thioflavin T fluorescence in the presence of saa3Dip (n=3), demonstrating lack of interaction between the inhibitor and the dye.

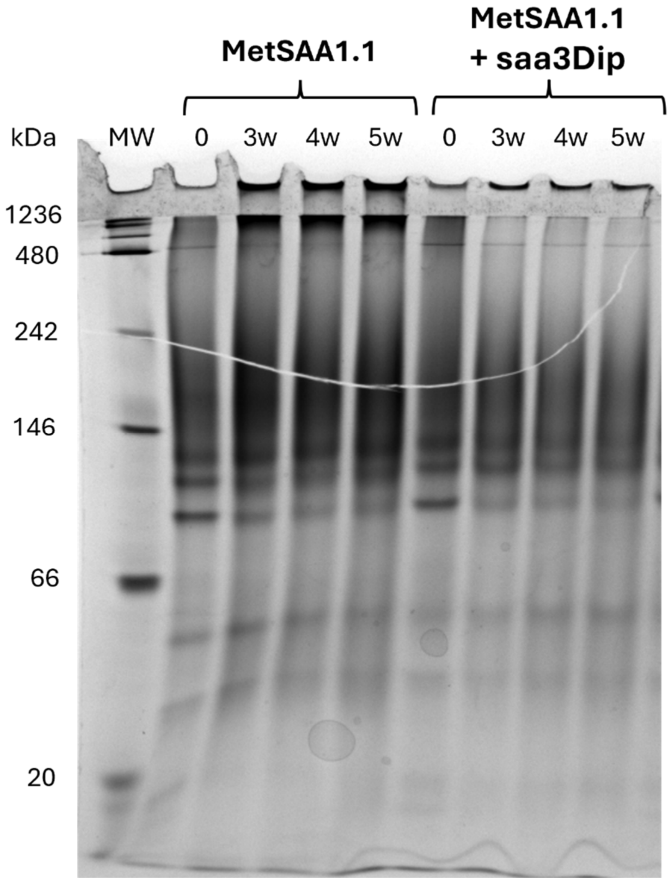

Figure S7. Electrophoregram of native PAGE for MetSAA1.1 and MetSAA1.1+saa3Dip samples incubated for 0, 3, 4 and 5 weeks at 37°C.

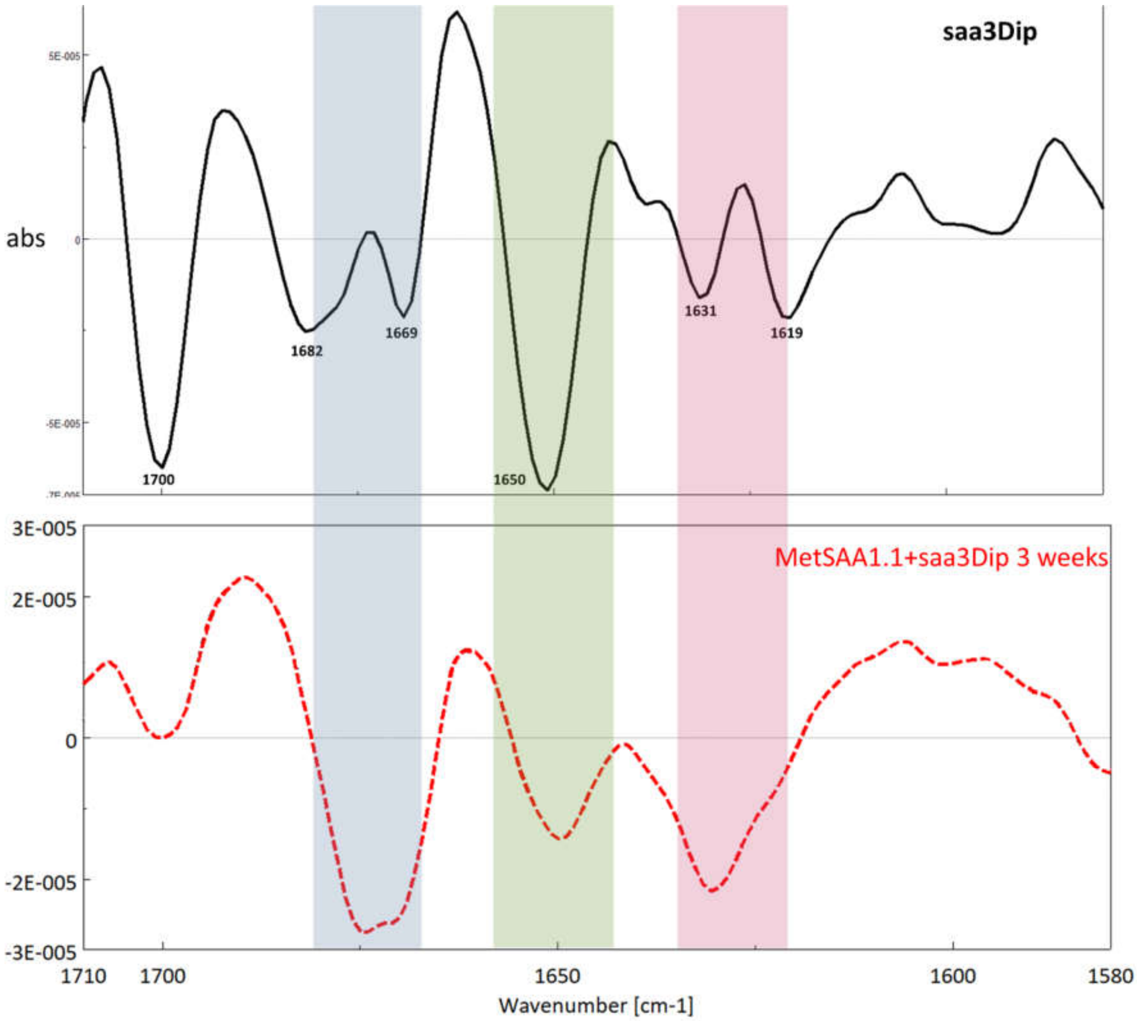

Figure S8. Second-derivative of the FTIR spectra in the amide I region of saa3Dip inhibitor and MetSAA+saa3Dip after 3 weeks of incubation at 37°C.

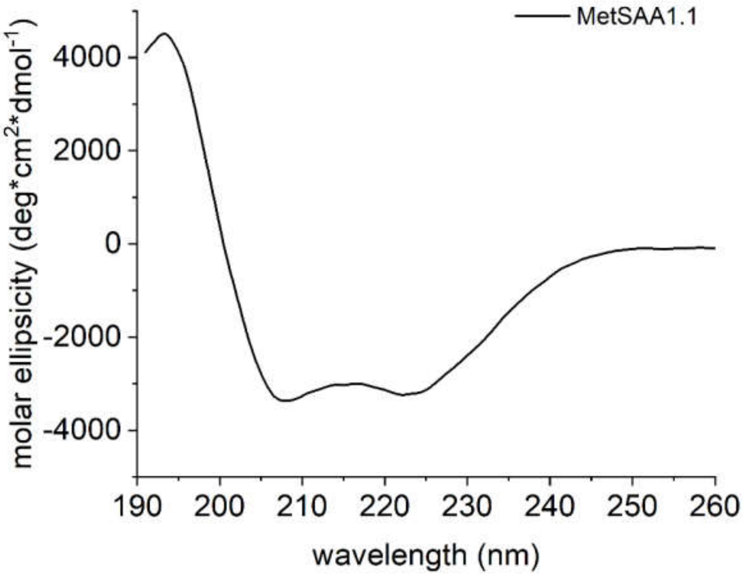

Figure S9. Far-UV Circular Dichroism spectrum of native MetSAA1.1.

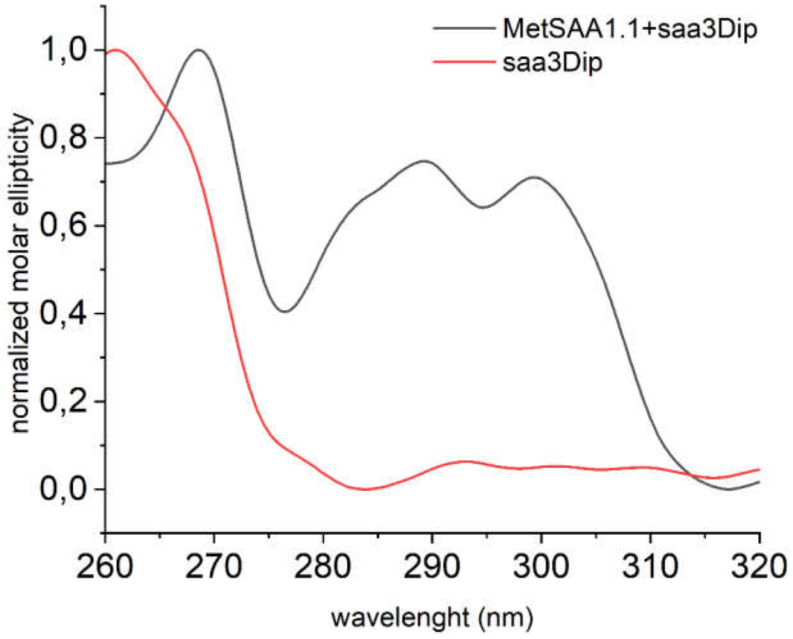

Figure S10. Circular Dichroism spectra (aromatic range) of MetSAA1.1 with saa3Dip, incubated for 3 weeks at 37°C (black line) and spectra of saa3Dip alone (red line).

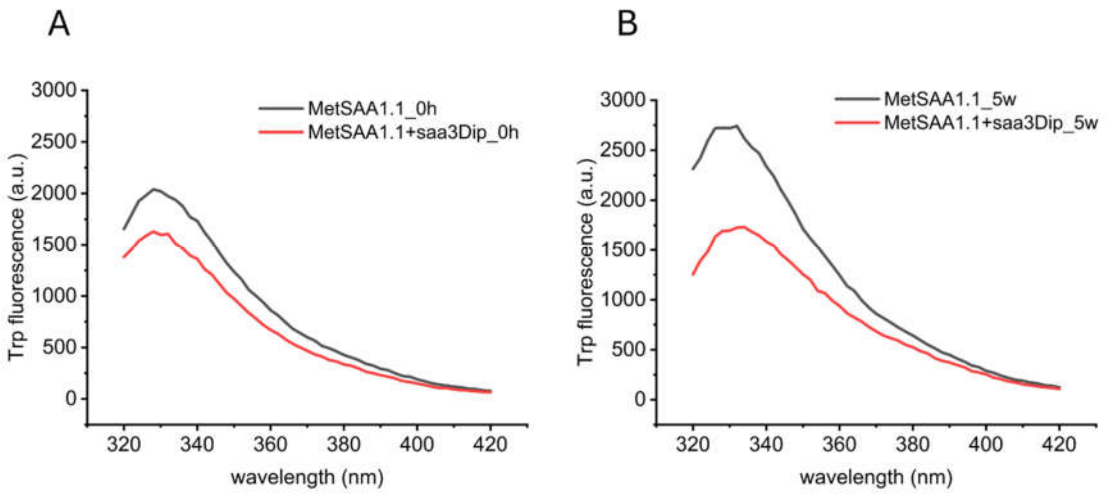

Figure S11. Representative spectra showing intrinsic Trp fluorescence of MetSAA1.1 and MetSAA1.1+saa3Dip: A) at the starting point and B) after 5 weeks of incubation at 37°C.

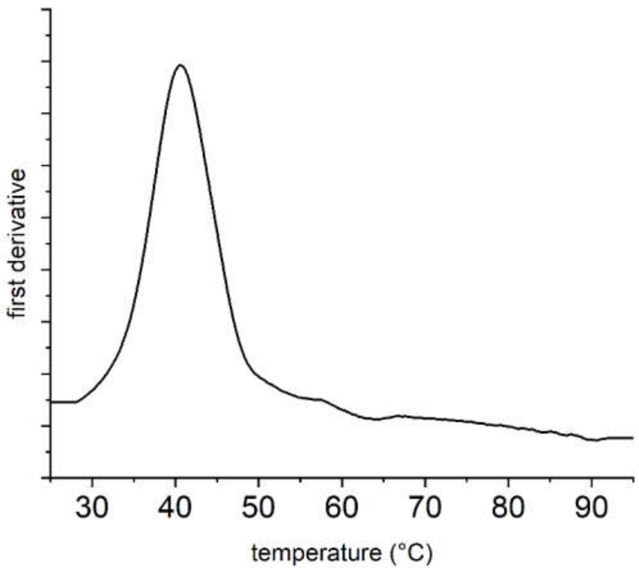

Figure S12. First derivative of thermal unfolding curve of MetSAA1.1 protein. The protein stability was observed using differential scanning fluorimetry instrument Prometheus NT48 (Nanotemper). The heating speed was 1.5°C/ 1 min. To determine the unfolding transition-point the tryptophan fluorescence at the emission wavelengths of 330 nm and 350 nm were recorded. The derivative was calculated from F350 nm / F330 nm ratio plotted as a function of temperature.
